# Decoding the genetic basis of demyelination: Prediction of potential pathogenic coding and regulatory noncoding MBP SNPs in multiple sclerosis

**DOI:** 10.1371/journal.pone.0347598

**Published:** 2026-05-21

**Authors:** Arwa Ibrahim Alwabran, Ghalia Mahfod Aldoseri, Ghanem Mahfod Aldoseri, Samah O. Mohager, Mobarak Mahfod Aldoseri, Ebtihal Kamal

**Affiliations:** 1 College of Medicine, Prince Sattam bin Abdulaziz University, Al-Kharj, Saudi Arabia; 2 Department of Basic Medical Sciences, College of Medicine, Prince Sattam bin Abdulaziz University, Al-Kharj, Saudi Arabia; Texas A&M University-San Antonio, UNITED STATES OF AMERICA

## Abstract

**Background:**

The Myelin Basic Protein (MBP) gene is essential for myelin sheath formation in the central nervous system. Coding and noncoding single-nucleotide polymorphisms (SNPs) can impair the protein structure and function, contributing to demyelinating diseases exemplified by multiple sclerosis. This study aimed to assess the impact of SNPs in the *MBP* gene on protein structure and function.

**Methods:**

We employed a comprehensive approach to investigate the impact of both noncoding and coding SNPs of the *MBP* gene. Initially, we utilized RegulomeDB to assess the regulatory roles of SNPs located in the 3′ untranslated regions (3′ UTRs). Subsequently, we examined the influence of the 3’ UTR SNPs on microRNA (miRNA) binding sites using PolymiRTS. Furthermore, we analyzed the functional 3′ UTR SNPs using RNAfold to evaluate their impact on RNA structure. To predict deleterious nonsynonymous SNPs (nsSNPs), various bioinformatics tools, including SIFT, PolyPhen-2, PROVEAN, META-SNP, ESNPs&GO, PANTHER, and AlphaMissense, were employed. Protein stability was assessed using I-Mutant2.0, MUpro, and DDMut. Structural modeling was performed with AlphaFold, and both wild-type and mutant proteins were visualized in UCSF ChimeraX. Conservation analysis was conducted using the ConSurf tool, and protein interaction networks were explored using the STRING database.

**Results:**

Eight noncoding SNPs were identified as potential regulatory SNPs, affecting the miRNA binding sites. Moreover, three nsSNPs, rs1971676214 (D173E), rs1242552448 (D173H), and rs772570115 (G176W), were consistently predicted to be pathogenic and to destabilize the protein structure. These variants were located in highly conserved sites and disrupted hydrogen bonds. STRING analysis revealed interactions between MBP and other myelin-related, immune, and signaling proteins, linking it to CNS and autoimmune pathways.

**Conclusions:**

This study identified eight noncoding 3′ UTR SNPs and three potentially pathogenic nsSNPs that may compromise gene expression and protein structure and function, respectively, offering insight into genetic mechanisms of demyelination.

## 1. Introduction

The Myelin Basic Protein (MBP) gene plays a critical role in the central nervous system (CNS) by encoding a protein that contributes to the assembly, compaction, and stabilization of the myelin sheath. This multilayered membrane protein insulates axons, enabling rapid saltatory nerve conduction and maintaining the efficiency of neural signaling [1, 2]. The *MBP* gene is located on chromosome 18q22 and spans approximately 45 kilobases, containing seven exons [3, 4]. Its expression is primarily restricted to oligodendrocytes within the CNS, although lower levels are also observed in Schwann cells of the peripheral nervous system [1]. MBP is characterized by its high basicity and intrinsically disordered structure, properties that facilitate its interaction with negatively charged membrane surfaces and cytoskeletal elements [2]. Through alternative splicing, the *MBP* gene generates multiple isoforms with distinct functional properties contributing to the structural integrity and plasticity of the myelin sheath [5]. Among these are the classic MBP isoforms predominant in the CNS and the Golli-MBP variants, which are expressed in immune-related tissues, notably within the thymus and spleen, suggesting additional immunological functions for this protein [6, 7]. Disruption of the *MBP* gene expression or its structural integrity has been closely linked to demyelinating diseases, particularly multiple sclerosis (MS), a chronic autoimmune disorder characterized by immune-mediated degradation of the myelin sheath, resulting in neurological dysfunction [8]. MBP is recognized as a major autoantigen in MS, with its degradation products frequently detected in the cerebrospinal fluid of individuals affected by the disease. Experimental studies have demonstrated that immunodominant fragments of MBP can provoke T-cell-mediated demyelination, emphasizing its pivotal role in the pathogenesis of MS [9]. The untranslated regions (UTRs) of a gene play an essential role in regulating its expression. 3′UTR governs mRNA stability, subcellular localization to myelin sheaths, and local translation in oligodendrocyte processes, largely through interactions with RNA-binding proteins and microRNAs (miRNAs) [10–12]. Experimental studies demonstrate that proper function of the MBP 3′ UTR is critical for mRNA transport and translational control in oligodendrocytes, with disruptions leading to altered protein expression [13]. SNPs in the UTR may therefore contribute to dysregulated MBP expression and increase susceptibility to demyelinating disorders. Nonsynonymous single-nucleotide polymorphisms (nsSNPs) represent the subset of coding variants that can directly alter amino acid sequence and protein function and represent the most prevalent form of genetic variation within the human genome. These variations have potential to substantially impact protein structure and function [14,15]. Several nsSNPs within the MBP gene have been associated with susceptibility to MS and variability in disease progression [8]. Despite these findings, the functional implications of numerous MBP nsSNPs remain largely unexplored, representing a significant gap in the understanding of the genetic basis of demyelinating disorders. Therefore, characterizing these genetic variations is essential for enhancing diagnostic, prognostic, and therapeutic strategies for demyelinating diseases. Bioinformatics provides a reliable, time-efficient, and cost-effective approach to predict the potential pathogenicity of SNPs by assessing their effects on gene expression, protein stability, structure, and interactions [16]. Computational analyses enable the prioritization of potential disease-causing variants for experimental validation, facilitating the identification of molecular targets implicated in disease development. In the present study, a comprehensive bioinformatics approach was applied to analyze both coding and 3′ UTR-noncoding SNPs within the MBP gene. The objective was to identify variants with potential effects on miRNA binding sites, protein structure, and function, thereby providing a foundation for future experimental research into the genetic mechanisms underlying myelin dysfunction and autoimmune neurodegeneration. To our knowledge, this study represents the first in silico investigation to systematically assess both noncoding and coding *MBP* variants using combined analyses of structural, regulatory, and stability effects.

## 2. Materials and methods

### 2.1. Analysis of MBP gene expression

Using the GTEx Database The Genotype-Tissue Expression (GTEx) database provides comprehensive transcriptomic data from a wide range of normal human tissues, enabling the assessment of tissue-dependent expression variability [17]. The GTEx database v8 (https://gtexportal.org/home/) was accessed on 29 September 2025 to analyze the tissue-specific expression profile of the *MBP* gene.

### 2.2. Protein information retrieval

Data for the human *MBP* gene were obtained from the National Center for Biotechnology Information (NCBI) website (https://www.ncbi.nlm.nih.gov/, accessed on May 14, 2025). Information on SNPs, including SNP IDs for the *MBP* gene, was retrieved from the NCBI dbSNP database (https://www.ncbi.nlm.nih.gov/datasets/gene/4155/) (accessed on 14 May 2025).

Protein sequence and annotation for MBP were obtained from the UniProt database (UniProtKB; https://www.uniprot.org), entry P02686, accessed on 14 May 2025. UniProt is a curated resource of protein sequences and functional information [18]. (https://www.uniprot.org/, accessed May 14, 2025).

### 2.3. Retrieval of UTR SNPs and annotation using regulomeDB

RegulomeDB v 2.2 (https://regulomedb.org/regulome-search/) is an integrative bioinformatics platform that systematically annotates non-coding UTR SNPs by consolidating high-throughput genomic, epigenomic, and transcriptional data from major consortia, including ENCODE and GEO. This database facilitates the functional interpretation of variants within regulatory elements, thereby identifying SNPs with potential roles in the modulation of gene expression. RegulomeDB assigns scores to SNPs to facilitate the identification of functional SNPs [19, 20]. The rank scores in RegulomeDB range from 1 to 7 (subcategorized into 1a–f, 2a–c, 3a, 3b, 4, 5, 6, and 7), wherein lower scores denote a higher likelihood of functional regulatory activity [21]. NCBI was accessed for the retrieval of 3′ UTR SNPs. Functional annotation and scoring of 3′ UTR SNPs of *MBP* were performed through RegulomeDB, and the scores of *MBP* non-coding SNPs were determined based on their corresponding rs identifiers. RegulomeDB was accessed on 2nd June 2025

### 2.4. Determination of 3′ UTR SNPs eQTLs

We used the GTEx database v8 to study the expression quantitative trait loci (eQTLs) of 3′ UTR SNPs,which were classified as functional in RegulomeDB. The portal identifies the associations between genetic variants and gene expression across tissues using a linear regression model implemented in the GTEx pipeline to test associations between variants and gene expression, adjusting for covariates specified by GTEx. Violin plots are generated to visualize gene expression levels across different genotypes of a specific variant within a selected tissue. The plot displays both the distribution and median expression for each genotype group, along with the corresponding p-value < 0.05, indicating the significant association. There is no additional multiple-testing correction performed in this study. GTEx portal accessed on 12 January 2026.

### 2.5. *The* functional impact *of* 3′ UTR SNPs *on* microRNA binding affinity

The correlation between 3′ UTR SNPs and miRNA binding sites was analyzed through the PolymiRTS Database v3.0 (https://compbio.uthsc.edu/miRSNP/). This computational resource systematically detects noncoding SNPs positioned within miRNA seed sequences or their complementary recognition sites. It predicts potential alterations in miRNA–mRNA interaction dynamics caused by these genetic variations [22]. miRNAs exert post-transcriptional control over gene expression by promoting translational inhibition or mRNA decay and sequence alterations within miRNA genes or their corresponding target regions. The PolymiRTS algorithm categorizes SNP-related effects into four distinct groups: D (loss of a conserved binding site), N (loss of a non-conserved site), C (emergence of a novel site), and O (undefined ancestral allele status). The polymiRTS database provides extensive insights into sequence conservation, ancestral versus derived alleles, and variations in context- dependent scores, thereby enabling a comprehensive evaluation of the functional regulatory implications of each identified 3′ UTR SNP. PolymiRTS Database was accessed on 25 June 2025

### 2.6. Effect of 3′ UTR SNPs on the secondary structure of mRNA

mRNA secondary structure predictions for 3′UTR variants were generated using RNAfold from the ViennaRNA Package v 2 (http://rna.tbi.univie.ac.at), accessed on 20 May 2025. RNAfold predicts minimum free-energy (MFE)structures and base-pairing probabilities for RNA sequences. It utilizes thermodynamic principles and dynamic programming to determine the most stable folding conformation. MFE postulates that an RNA molecule folds into the structure that minimizes its overall Gibbs free energy (ΔG) under defined conditions [23]. The 3′ UTR SNPs annotated with RegulomeDB scores of ≤2b, indicating potential disruption or facilitation of novel miRNA binding sites, were prioritized for downstream analysis using the RNAfold server. The MFE values and the corresponding mRNA secondary structures were calculated using wild-type and variant sequences obtained from NCBI to evaluate the structural impact of each 3′ UTR SNP. RNAfold was accessed on 5 July 2025.

### 2.7. Assessment of deleterious nsSNPs and their predicted phenotypes

To predict deleterious nsSNPs, seven different computational tools were employed. The bioinformatics tools used in the prediction of the nsSNPs effect, and their cutoff Values were summarized in Table 1.

**Table 1:** Summary of the bioinformatics tools, Cut-Off Values and accession dates.

Bioinformatics Tool/Server and versions	Cut-Off Value	Accession dates
SIFT v 5.2.2	Tolerance Index (TI) < 0.05 indicates deleterious. ≥ 0.05 indicates tolerated [24,25]	Accessed 14 May 2025
PolyPhen-2 v 2.2.2	A score ≥ 0.85 indicates probable damage. score > 0.5 < 0.85 possibly damaging < 0.5 benign [26]	Accessed 14 May 2025
PROVEAN v 1.1	Score ≤ −2.5 indicates deleterious. A score > −2.5 indicates benign [27]	Accessed 16 May 2025
META-SNP web server (build 2014_01)	A consensus score > 0.5 indicates pathogenic nsSNPs [28].	Accessed 18 May 2025
ESNPs&GO 2017 web server release	RI closer to 0 indicating benign variants, RI closer to 10 indicating pathogenic variants [29]	Accessed 22 May 2025
PANTHER v17.0, PSEP module	Probably damaging (PSEP time > 450 ms), possibly damaging (PSEP 450 my > time> 200 ms), and probably benign (PSEP time < 200 my [30]	Accessed 23 May 2025
Alpha Missense v1 scores (2023 release)	Pathogenicity score: 0–0.34 likely benign, 0.34–0.564: ambiguous,0.564–0.78: likely pathogenic 0.78–1.0 [31]	Accessed 6 July 2025
I-Mutant 2.0	ΔΔG > 0 indicates decreased stability [32]	Accessed 27 May 2025
MUpro web server v1.0	Score < 0.0 indicates a destabilizing effect. Score > 0.0 indicates stabilizing effect [33]	Accessed 28 May 2025).
DDMut web server (2021 release)	negative ΔΔG are labeled destabilizing, while those with positive ΔΔG are labeled stabilizing [34]	Accessed 19 May 2025
ConSurf 2023 server	Conservation scores 9 indicates the highest conservation [35]	Accessed 9 July 2025
AlphaFold DB model AF-P02686-F1, version 4	pLDDT > 90 Very high confidence, 90 > pLDDT > 70 High confidence, 70 > pLDDT > 50 Low confidence, pLDDT < 50 Very low confidence [36]	Accessed 2 June 2025
STRING v12.0	Interaction score > 0.4 indicates significant interaction [37]	Accessed 2 June 2025

*∆∆G (changes in Gibbs free energy); PSEP (position-specific evolutionary preservation); pLDDT (a per residue measure of local confidence)*.

Sorting Intolerant From Tolerant (SIFT) (http://sift.bii.a-star.edu.sg/,) predicts the impact of amino acid substitutions on protein function. The nsSNP IDs were collected from NCBI databases and submitted to SIFT for analysis. nsSNPs with scores ranging from 0.0 to 0.04 were classified as damaging, whereas those with scores from 0.05 to 1.0 were considered benign [25]. PolyPhen 2.0 (http://genetics.bwh.harvard.edu/pph2/,). It evaluates the potential impact of nsSNPs by analyzing sequence conservation, structural features, and functional domains of the protein. The tool categorizes nsSNPs as “benign,” “possibly damaging,” or “Probably damaging” based on their likelihood to affect protein function [26]. Deleterious nsSNPs predicted in SIFT were used as input in PolyPhen 2.0 to assess the possible effects of amino acid substitutions on protein function. Protein Variation Effect Analyzer (PROVEAN) (https://www.jcvi.org/research/provean/,) is a computational too used to predict the effect of amino acid substitutions or insertions/deletions on protein function. Variants scoring −2.5 or lower are classified as deleterious, while those with scores above −2.5 are considered neutral [27]. To evaluate the potential pathogenicity of the selected nsSNPs, we utilized the META-SNP tool (https://snps.biofold.org/meta-snp/pages/methods.html,), a consensus- based web server that integrates the outputs of several individual nsSNP effect prediction tools [28]. The ESNPs&GO tool (https://esnpsandgo.biocomp.unibo.it/,) was used to predict the pathogenicity of nsSNPs. This method integrates protein sequence embeddings generated by advanced protein language models (ProtTrans T5 and Anc2Vec) along with Gene Ontology (GO) annotations to assess the functional impact of amino acid substitutions. The tool applies a support vector machine (SVM) classifier to provide a probability score indicating whether a variant is likely disease-associated or neutral [29]. The Protein Analysis Through Evolutionary Relationships (PANTHER) tool (http://pantherdb.org/,) evaluates evolutionary conservation using the PANTHER PSEP (Position-Specific Evolutionary Preservation) module, which was used for variant effect prediction, rather than the broader PANTHER database for GO enrichment. The PSEP estimates the evolutionary preservation time of amino acid positions to infer variant impact [30]. Based on the PSEP value, nsSNPs are categorized as “probably benign” if conserved for less than 200 million years, “possibly damaging” if less than 450 million years but more than 200 million years, and “probably damaging” if conserved for 450 million years or more, according to the PANTHER-PSEP server’s default classification.

### 2.8. Computational prediction *of* missense variant pathogenicity using AlphaMissense

AlphaMissense (https://alphamissense.hegelab.org/search,) is a deep learning-based method, designed to classify nsSNPs as benign or pathogenic by integrating structural and functional features of proteins for cell survival. The tool provides pathogenicity scores ranging from 0 to 1, where high scores indicate a higher probability of pathogenicity. Based on the generated scores, nsSNPs were classified as likely pathogenic or likely benign [31]. We used AlphaMissense to predict the pathogenicity of missense variants in the *MBP* gene.

### 2.9. Effect of MBP nsSNPs on protein stability

I-Mutant 2.0 (https://folding.biofold.org/i-mutant/i-mutant2.0.html), a neural network-based tool that was used to predict the effect of nsSNPs on protein stability by estimating changes in ∆∆G. Protein sequences were input that we used to generate stability change predictions with associated confidence scores [32]. MUpro (http://mupro.proteomics.ics.uci.edu), a suite of machine-learning techniques designed to assess the effect of individual nsSNPs on protein stability. It combines SVM and neural networks to classify whether stability increases or decreases and provides a ∆∆G prediction with an associated confidence score ranging from −1–11 [33]. DDMut (https://biosig.lab.uq.edu.au/ddmut/,) utilizes deep learning method to rapidly and accurately predict changes in ∆∆G for both single and multiple mutations [34].

### 2.10. Population allele frequency and clinical annotation analyses

To study the Population allele frequency, we used the gnomAD v4.1.0 (https://gnomad.broadinstitute.org/). The input was the gene name *MBP,* and global allele frequencies and functional annotations of all *MBP* variants [38]. Clinical annotations for *MBP* variants were checked using the ClinVar database (https://www.ncbi.nlm.nih.gov/clinvar/), a weekly updated public archive of interpretations of the clinical significance of human genetic variants, aggregated from multiple clinical and research submitters analysis [39]. GnomAD and ClinVar were accessed on 10 January 2026.

### 2.11. Computational analysis *of* evolutionary conservation

We utilized the ConSurf server (https://consurf.tau.ac.il,) to analyze the evolutionary conservation of nsSNPs within the protein sequence. This analysis identified highly conserved residues and categorized them as either exposed or buried [35]. The FASTA sequence of MBP was submitted to the ConSurf server to quantitatively assess the conservation levels of individual amino acid residues.

### 2.12. Variant prioritization criteria

NsSNPs were prioritized using a multi-step in silico filtering strategy integrating functional, stability, and evolutionary evidence. First, all *MBP* nsSNPs retrieved from dbSNP were screened with SIFT, and nsSNPs with scores ≤ 0.04 were classified as deleterious and retained for further analysis, whereas those with scores ≥ 0.05 were considered tolerated and excluded. Next, the SIFT- positive variants were evaluated with PolyPhen-2 and PROVEAN; only nsSNPs predicted as “probably/possibly damaging” by PolyPhen-2 and “deleterious” by PROVEAN (score ≤ −2.5) were considered for further analysis. To refine pathogenicity, the predicted deleterious nsSNPs were subsequently analyzed using META-SNP, ESNPsGO, PANTHER-PSEP, and AlphaMissense.

Only nsSNPs consistently classified as disease-associated or likely pathogenic across these tools were retained. nsSNPs meeting these functional/pathogenic criteria were then subjected to stability prediction tools. NsSNPs predicted to decrease stability by the three tools (I-Mutant 2.0, MUpro, and DDMut) were prioritized as stability-impairing nsSNPs. nsSNPs located at positions with the highest conservation scores and classified as buried structural residues predicted in ConSurf were considered for downstream structural and interaction analyses.

### 2.13. Computational prediction and visualization of protein structure

We used AlphaFold (https://alphafold.ebi.ac.uk,), an advanced artificial intelligence system developed by Google DeepMind, to predict the three-dimensional structure of the MBP. AlphaFold applies deep learning to accurately model protein structures from their amino acid sequences [36]. The UniProt sequence of MBP was used as input to generate the structural model. To visualize and analyze the predicted structure, we employed UCSF ChimeraX 1.9 (https://www.rbvi.ucsf.edu/chimerax/, accessed on 2 June 2025), a versatile molecular visualization tool that supports the interactive examination of structural data, including density maps, sequence alignments, and large biomolecular complexes [40]. ChimeraX was used to map and compare amino acid substitutions between the wild-type and mutant forms of MBP.

### 2.14. Prediction of changes in the biological interactions between wild- type and mutant MBP protein

We used DDMut to detect the changes in the biological interactions between wild-type amino acids and neighborhood residues in comparison with mutant residues [34].

### 2.15. Estimation of protein-protein interactions

We used the STRING database, version 12.0 (https://string-db.org/,), to study MBP-protein interactions. This database integrates data from experiments, computational predictions, and text mining and assigns a confidence score to each interaction [37, 41]. We used MBP as the input in the protein-by-name module, selecting “Homo sapiens” as the species. Furthermore, we configured the following parameters: meaning of network edges: “evidence,” active interaction sources: “Experiments, Text Mining, Databases, expression, Neighborhood, and co-occurrence,” The minimum required interaction score is “medium confidence 0.700,” and the maximum number of interactors to display is “no more than 50 interactors.” Finally, the MBP- interacting proteins were visualized. Gene Ontology (GO) enrichment analysis for MBP-related proteins were performed using STRING. Enriched biological process, molecular function, and cellular Component terms were identified based on the statistics provided by STRING database. GO Terms with a false discovery rate (FDR) < 0.05 were considered more robust signals and considered significantly enriched.

## 3. Results

### 3.1. Tissue-specific expression analysis of MBP using GTEx

**Fig 1:**
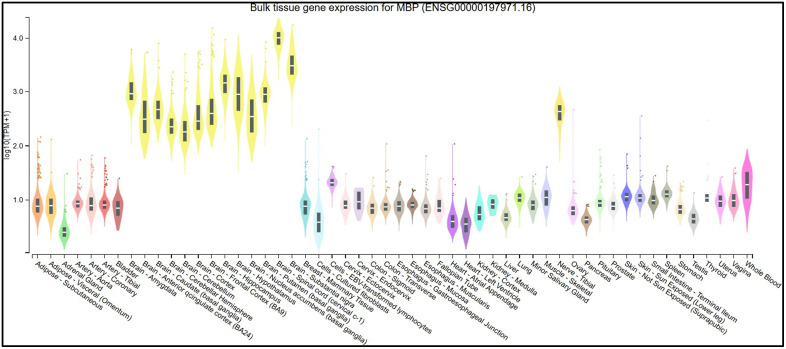
Expression Analysis of MBP across different tissues.

GTEx Expression analysis showed MBP is predominantly expressed in brain tissues, with the highest expression detected in the brain white matter, cerebellum, anterior cingulate cortex (BA24), and frontal cortex (BA9). Conversely, non-neural tissues, including adipose, liver, and blood, displayed minimal expression (Fig 1).

### 3.2. Distribution of MBP gene SNP datasets

**Fig 2:**
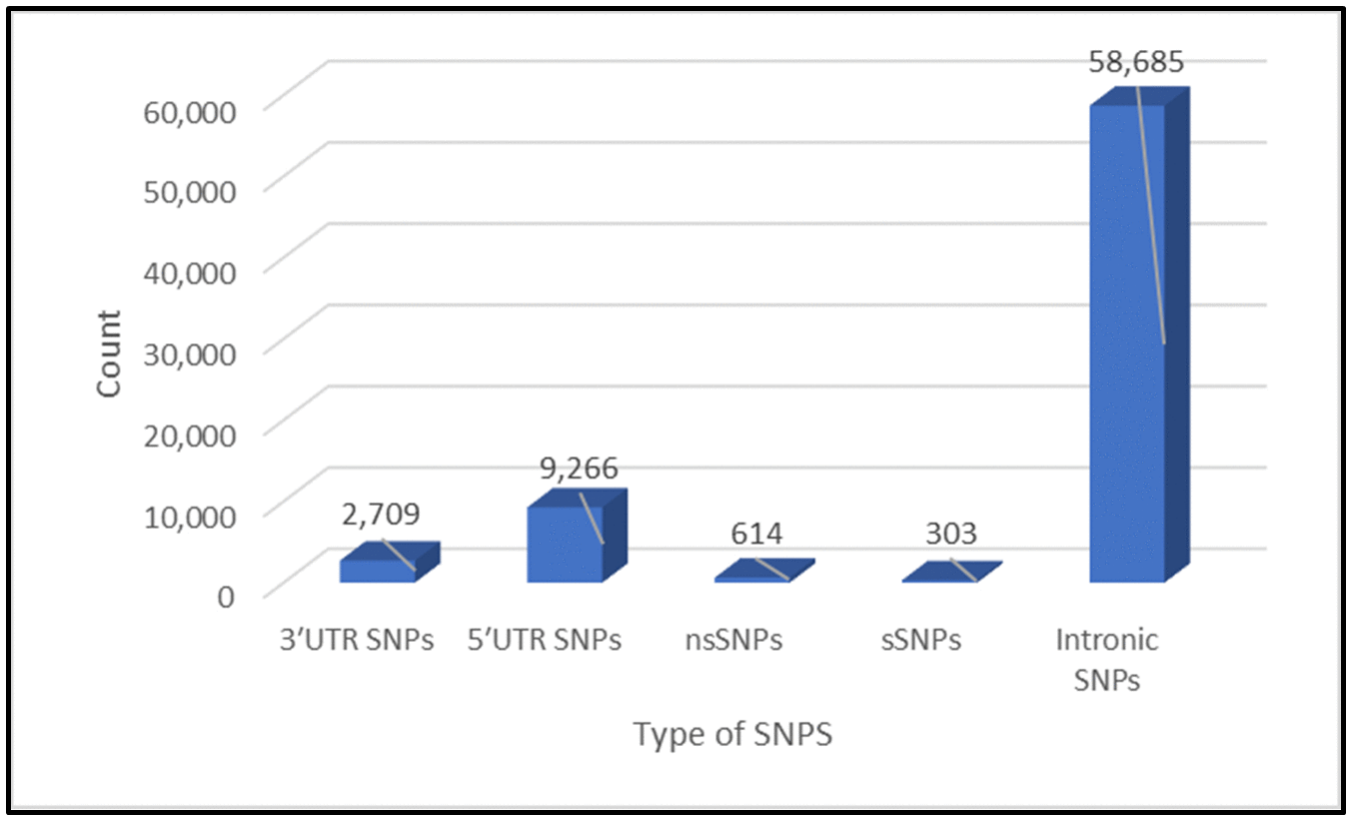
Distribution of SNPs within the *MBP* gene.

SNPs were retrieved from NCBI In the noncoding regions; there were 2,709 within the 3′UTR (S1 Table) and 9,266 within the 5′UTR. In addition, we found 614 were nsSNPs (S2 Table), 303 were synonymous SNPs (sSNPs), and 58,685 were within intronic sequences (Fig 2). For this study, we further analyzed both noncoding 3′UTR SNPs and nsSNPs.

### 3.3. Scoring of 3ˊUTR SNPs on RegulomeDB

**Fig 3:**
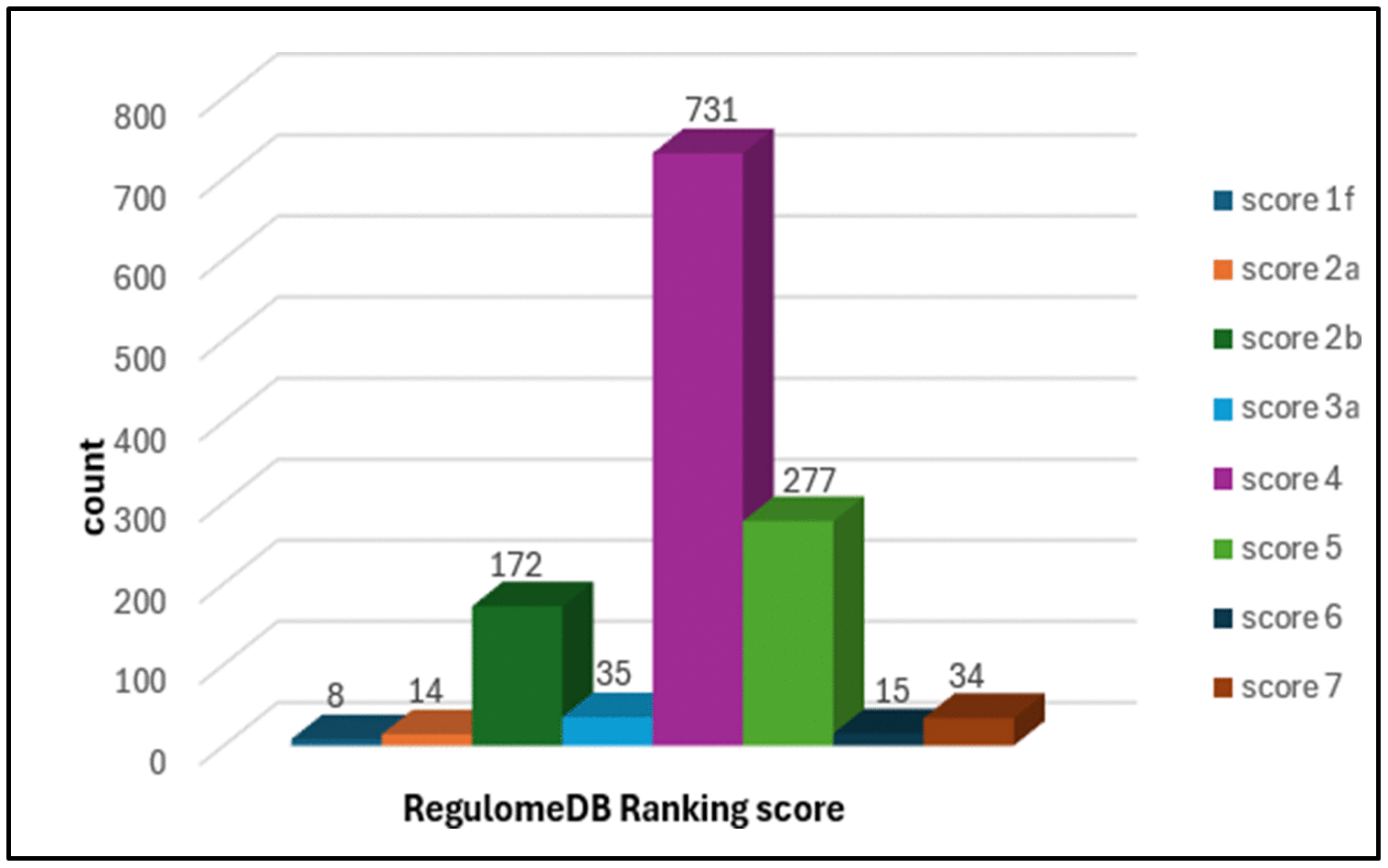
Annotation and scoring of MBP 3′UTR SNPs by RegulomeDB. *The plot shows the distribution of RegulomeDB scores for 1,286 MBP 3′UTR SNPs retrieved from the database. Lower scores (1f–2b) indicate stronger evidence for regulatory function, integrating transcription factor binding, DNase hypersensitivity, and chromatin state annotations, whereas higher scores (5–7) suggest minimal regulatory evidence. SNPs with scores of ≤ 2b were selected as functionally relevant for downstream analyses*.

The SNPs in the 3ˊUTR were subjected to functional evaluation through the RegulomeDB server. Out of all SNPs, 1,286 in the 3ˊ UTR were found on RegulomeDB (S3.1–S3.14 Table). A sum of 194 SNPs in 3ˊ UTR were annotated as ≤ 2b by RegulomeDB, having variable RegulomeDB scores (Fig 3).

### 3.4. Determination of 3ˊUTR SNPs eQTLs of MBP

**Fig 4:**
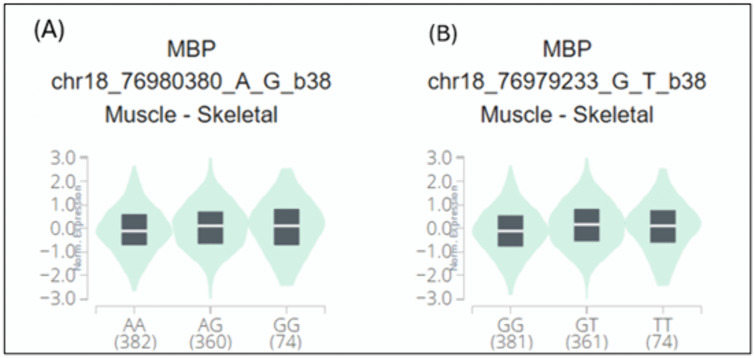
Violin plots showing eQTL effects of 3′ UTR SNPs on MBP expression in skeletal muscle: *Violin plots show normalized MBP expression across genotypes for (A) rs9199 (chr18_76980380_A_G_b38) and (B) rs1048947 (chr18_76979233_G_T_b38) in Muscle – Skeletal tissue*. *The y-axis represents normalized expression values (unitless), and the black boxes indicate the median and interquartile range for each genotype group. Sample sizes for each genotype are shown in parentheses below the x-axis labels (AA/AG/GG for rs9199 and GG/GT/TT for rs1048947). Associations were considered at nominal p < 0.05 and are interpreted as exploratory eQTL signals*.

All SNPs located within the 3′ UTRs of the *MBP* gene and classified as functional SNPs in RegulomeDB were evaluated for eQTL effects using the GTEx portal. GTEx v8 skeletal muscle eQTLs for *MBP* were derived from Muscle – Skeletal RNA-seq samples (n = 816 donors), and associations were considered significant at a nominal p < 0.05 as FDR/q-values were not used for filtering. Among all evaluated 3ˊUTR variants, two SNPs, rs9199 and rs1048947, located in the 3′ UTR, had significant association with *MBP* gene expression in skeletal muscle tissue (Fig 4). The normalized effect size (NES) indicated regulatory effects on gene expression with p-values of 0.0000067 and 0.000020, respectively (Table 2).

**Table 2:** Result of eQTLs analysis through GTEx of rs9199 and rs1048947.

	Rs SNP ID	P-value	NEZ	Tissue
1	rs9199	0.0000067	0.11	Skeletal muscle
2	rs1048947	0.000020	0.11	Skeletal muscle

### 3.5. Association *of* 3’ UTR SNPs *with* miRNAs

The impact of 3’ UTR SNPs, as retrieved from NCBI, on miRNA was evaluated using PolymiRTS. It identified the association of SNPs with miRNAs, along with functional impact scores. 147 SNPs were identified as disrupting the conserved region of miRNA (D score), with scores ranging from 2 to 10. Additionally, 121 SNPs facilitated the formation of de novo miRNA-binding sites, as indicated by the C score (S4 Table).We identified eight functional 3’UTR SNPs with scores ≤ 2b that either disrupt or create novel miRNA binding sites. The RegulomeDB and PolymiRTS scores are presented in Table 3.

**Table 3:** RegulomeDB and PolymiRTS scores of the functional 3′ UTR SNPs.

	Rs SNP ID	RegulomeDB	PolymiRTS
1	rs7236968	2b	C
2	rs62105705	1f	D
3	rs112470302	2b	C
4	rs117862091	2b	C
5	rs147599766	2b	C
6	rs4494640	2b	D
7	rs9959403	2b	C
8	rs142836347	2b	D

### 3.6. Impact *of* 3ˊUTR SNPs *on* secondary structure *of* mRNA

**Fig 5:**
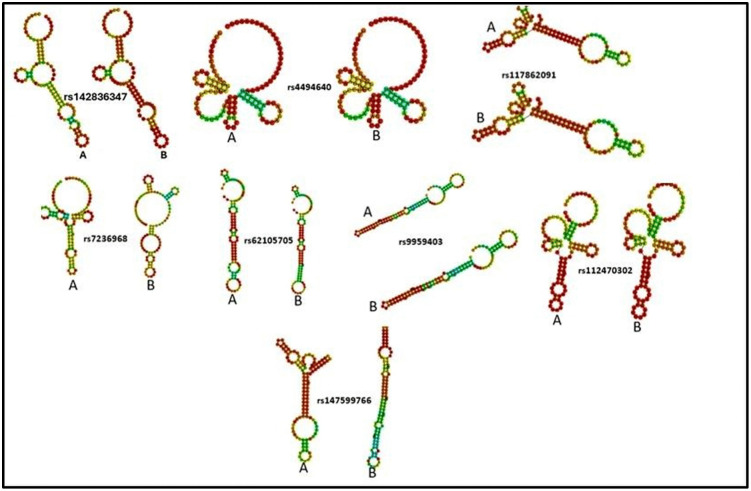
Predicted secondary structures of wild-type (A) and mutant (B) mRNA of 3′UTR SNP in the *MBP* gene generated using RNAfold. *The colour gradient represents the base-pairing probability, where red indicates highly stable paired regions, and green to yellow denotes less stable or unpaired bases*.

Eight functionally relevant 3ˊUTR SNPs identified by RegulomeDB (rank ≤ 2b), which either disrupt or generate novel miRNA binding sites, were selected for further structural analysis using RNAfold to illustrate potential structural differences between alleles, and no inferential statistics were applied. Computational predictions revealed that six of the 3′ UTR variants induced alterations in the MFE and secondary structure of the mRNA, whereas the remaining two SNPs produced negligible changes in both MFE (Table 4) and mRNA conformation (Fig 5).

**Table 4:** Minimum free energy (MFE) of wild and mutant structures of mRNAs caused by 3′ UTR SNPs of MBP.

	Rs SNP ID	MFE Wild (kcal/mol)	MFE Mutant (kcal/mol)	Interpretation
1	rs7236968	−7.30	−6.00	Energy is increased, destabilizing the mRNA structure
2	rs62105705	−13.90	−14.00	There is decrease in MFE in the mutant mRNA, causing structural change in mRNA and stabilizing the structure
3	rs112470302	−22.50	−22.50	No variation in energy
4	rs117862091	−31.00	−30.70	Energy is increased, destabilizing the mRNA structure
5	rs147599766	−32.60	−30.00	Energy is increased, destabilizing the mRNA structure
6	rs4494640	−11.60	−12.50	There is decrease in MFE in the mutant mRNA, causing structural change in mRNA and stabilizing the structure
7	rs9959403	−22.70	−22.70	No variation in energy
8	rs142836347	−21.20	−24.00	There is decrease in MFE in the mutant mRNA, causing structural change in mRNA and stabilizing the structure

A total of 614 nsSNPs were initially extracted and analyzed using multiple in silico pathogenicity prediction tools. Among these, 218 variants were identified as potentially deleterious by SIFT (S5 Table) and were subsequently evaluated using two additional tools: PolyPhen-2 and PROVEAN. From this analysis, 172 nsSNPs were classified as damaging in PolyPhen-2 (S6 Table). 99 nsSNPs were predicted to be damaging by all three tools (S7 Table). These 99 potentially deleterious nsSNPs were then subjected to a second set of tools using META-SNP, ESNPs&GO, and PANTHER to assess their potential disease associations. We found that 54 nsSNPs were classified as disease-related by META-SNP (S8 Table), 17 nsSNPs by ESNPs&GO (S9 Table), and 9 by PANTHER. Ultimately, nine nsSNPs were consistently predicted as pathogenic across all six bioinformatics tools (Table 5). Further computational analyses were performed to evaluate the structural and functional consequences of these potentially disease-causing nsSNPs.

**Table 5:** List of nsSNPs predicted to be pathological by META-SNP, ESNP&GO, and PANTHER.

	Rs nsSNP ID	Amin Acid Change	META-SNP		ESNP& GO		PANTHER	
			Prediction	Score	Prediction	RI	Effect	Preservation Time
1	rs1971676214	D173E	Disease	0.672	Pathogenic	2	Probably damaging	455
2	rs1242552448	D173H	Disease	0.734	Pathogenic	6	Probably damaging	455
3	rs1568292714	G176E	Disease	0.721	Pathogenic	1	Probably damaging	455
4	rs772570115	G176R	Disease	0.737	Pathogenic	4	Probably damaging	455
5	rs772570115	G176W	Disease	0.803	Pathogenic	9	Probably damaging	455
6	rs748450291	R177C	Disease	0.676	Pathogenic	4	Probably damaging	455
7	rs779353840	R177H	Disease	0.646	Pathogenic	9	Probably damaging	455
8	rs1488047563	S174F	Disease	0.649	Pathogenic	6	Probably damaging	455
9	rs1194494874	S174P	Disease	0.691	Pathogenic	7	Probably damaging	455

### 3.8. Pathogenicity assessment of MBP nsSNPs using AlphaMissense

AlphaMissense analysis was performed to predict the pathogenicity of selected *MBP* gene variants. The results indicated that seven out of the predicted nine deleterious nsSNPs, including the rs1971676214 (D173E), rs1242552448 (D173H), rs772570115 (G176W), rs772570115 (G176R), rs1488047563 (S174F), and rs1194494874 (S174P), showed pathogenicity scores > 0.78 and were classified as likely pathogenic. In contrast, the rs779353840 (R177H) had a pathogenicity score of 0.181, indicating that it is likely benign. One variant, rs748450291 (R177C), was classified as ambiguous with pathogenicity scores of 0.38 (Table 6).

**Table 6:** AlphaMissense-Based Pathogenicity Assessment of Selected nsSNPs.

	RS nsSNP ID	Amino Acid Change	Pathogenicity	Class
1	rs1971676214	D173E	0.799	Likely Pathogenic
2	rs1242552448	D173H	0.917	Likely Pathogenic
3	rs1568292714	G176E	0.926	Likely Pathogenic
4	rs772570115	G176R	0.889	Likely Pathogenic
5	rs772570115	G176W	0.943	Likely Pathogenic
6	rs748450291	R177C	0.38	Ambiguous
7	rs779353840	R177H	0.181	Likely benign
8	rs1488047563	S174F	0.774	Likely Pathogenic
9	rs1194494874	S174P	0.916	Likely Pathogenic

### 3.9. Result of the impact of the deleterious nsSNPs on MBP protein stability

We utilized three in-silico prediction tools: I-MUTANT 2.0, MUpro, and DDMUT. The computational results demonstrated that nsSNPs rs1971676214 (D173E), rs1242552448 (D173H), and rs772570115 (G176W) exhibited a destabilizing effect on the MBP protein structure. These substitutions were consistently predicted to reduce protein stability, as evidenced by negative ΔΔG (kcal/mol) values across all tools (Table 7).

**Table 7:** *MBP* nsSNPs predicted to significantly impair protein stability based on I-MUTANT 2.0, MUpro, and DDMut computational analyses.

	RS nsSNP ID	Amino Acid change	I-Mutant2.0			MUpro		DDMut	
			Stability	RI	DDG (kcal/mol)	Stability	DDG (kcal/mol)	Stability	DDG (kcal/mol)
1	rs1971676214	D173E	Decrease	1	−1.7	Decrease	−0.56816813	Destabilizing	−0.48
2	rs772570115	G176W	Decrease	8	−2.58	Decrease	−0.99465147	Destabilizing	−0.03
3	rs1242552448	D173H	Decrease	6	−2.08	Decrease	−0.93017763	Destabilizing	−1.34

### 3.10. Results *of* population allele frequency and clinical annotation analyses

To study the Population allele frequency, we used the gnomAD database. The input was the gene name *MBP,* and global allele frequencies and functional annotations of all *MBP* SNPs were retrieved (S10 Table). We found that one prioritized *MBP* nsSNP, rs1971676214 (D173E), was extremely rare in the global population, including African, Latino/Admixed American, East Asian, South Asian, European (non-Finnish and Finnish), and Middle Eastern cohorts, consistent with a very rare variant. Rs1242552448 (D173H) and rs772570115 (G176W) were not reported in the gnomAD database. No ClinVar records with definitive pathogenic or likely pathogenic classifications were identified for the potentially deleterious *MBP* nsSNPs. These nsSNPs were unclassified and remain unannotated in the current clinical databases, indicating that their predicted deleterious effects on MBP structure and function have not yet been captured in routine clinical variant interpretation workflows.

### 3.11. Result of conservation analysis

The evolutionary conservation of amino acid residues in the MBP protein was evaluated using the ConSurf server. Three nsSNPs, rs1971676214 (D173E), rs1242552448 (D173H), and rs772570115 (G176W), were identified in highly conserved regions, with a conservation score of 9, the highest on ConSurf’s scale. Based on structural context, D173E, D173H, and G176W were predicted as structural residues, characterized by both high conservation and burial within the protein core (Table 8).

**Table 8:** Evolutionary Conservation Profiles of the Most Pathogenic nsSNP.

	RS nsSNP ID	Amino Acid Change	Conservation Score	Prediction
1	rs1971676214	D173E	9	Structural residue (highly conserved and buried)
2	rs772570115	G176W	9	Structural residue (highly conserved and buried)
3	rs1242552448	D173H	9	Structural residue (highly conserved and buried)

### 3.12. Protein 3D structure prediction by AlphaFold and nsSNPs visualization by Chimera X

**Fig 6:**
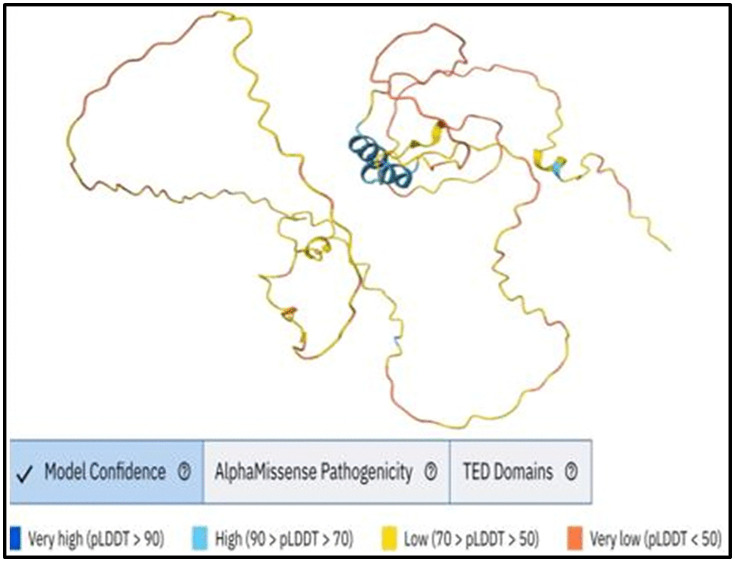
Protein 3D structure of human MBP predicted by AlphaFold.

**Fig 7:**
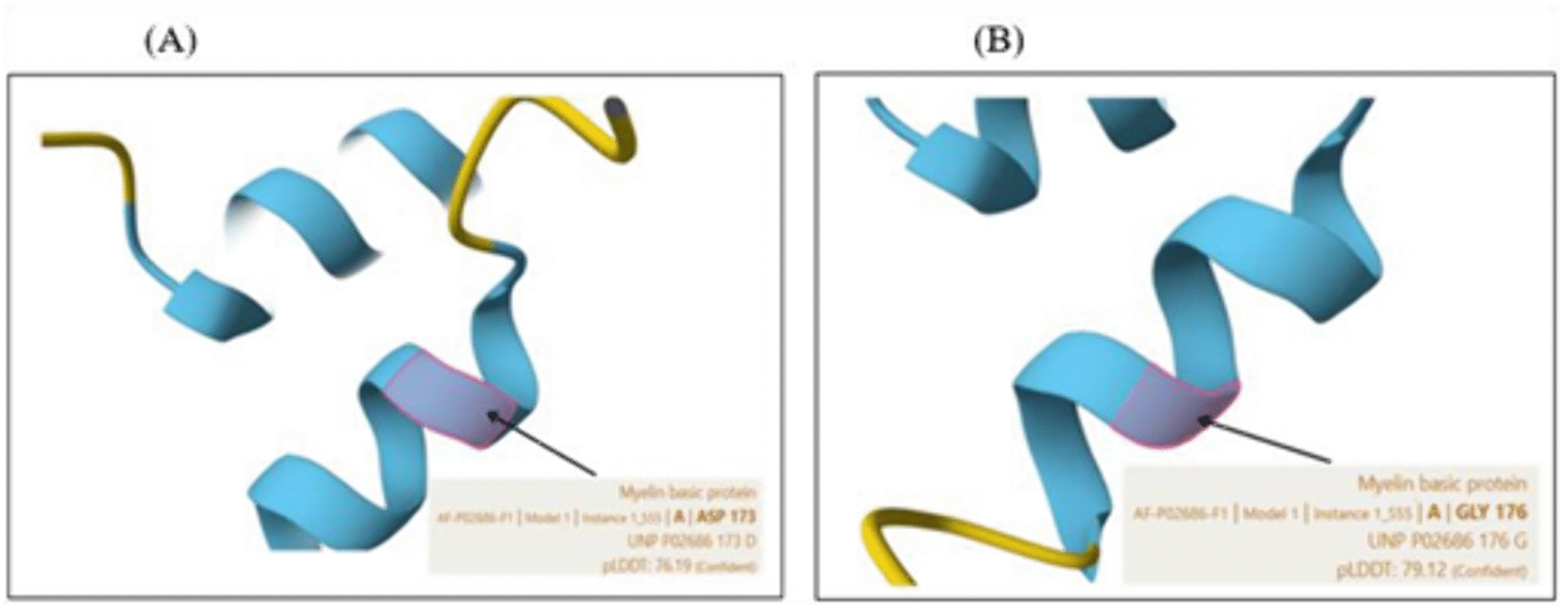
pLDDT scores of positions D173 (A) and G176 (B).

**Fig 8:**
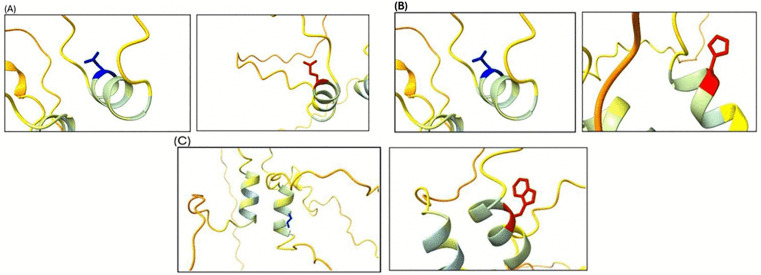
Effect of the three most deleterious nsSNPs on the MBP protein structure. (A)wild type amino acid at position 173(D)(left), mutant type amino acid at position 173(E)(right), (B)wild-type amino acid at position 173(D)(left), mutant-type amino acid at position 173(H)(right), (C)wild type amino acid at position 176 (G)(left)mutant type amino acid at position 176(W)(right).

The three-dimensional structure of the human MBP protein was predicted using the AlphaFold algorithm (Fig 6), which provides a confidence score called pLDDT (predicted Local Distance Difference Test) ranging from 0 to 100. Regions with pLDDT scores above 90 are considered highly confident, while other areas show moderate confidence (scores between 70 and 90), low confidence (50–70), or very low confidence (below 50). We studied the AlphaFold structural model of MBP. We found that positions D173 and G176 had predicted confident pLDDT scores of 76.19 and 79.12, respectively, indicating a good backbone prediction and a reliable backbone and side chain (Fig 7). The pdb structure files of the wild type (AF-P02686-F1-model_v6.pdb) and mutant proteins (D173E.pdb, D173H.pdb, and G176W.pdb) were provided in the supplementary files (S1 File for the wild-type protein, S2 File for D173E, S3 File for D173H, and S3 File for G176W). ChimeraX was used to visualize the 3D structures, displaying wild-type amino acids and the mutant residues (Fig 8).

### 3.13. Structural impact of predicted variants on hydrogen bonds

**Fig 9:**
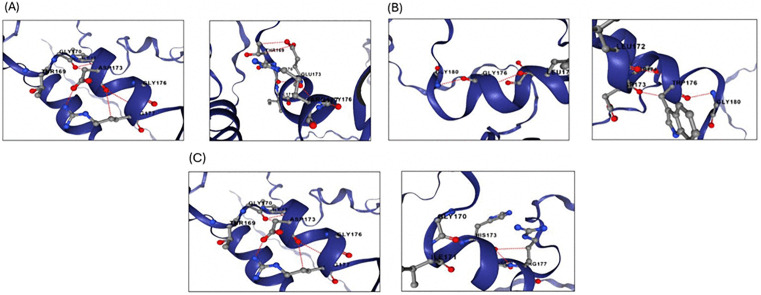
Difference in ionic interactions of the three most deleterious nsSNPs on the *MBP* protein between the wild-type and mutant residues. (A)wild type amino acid at position 173(D)(left), mutant type amino acid at position 173(E)(right), (B)wild-type amino acid at position 173(D)(left), mutant-type amino acid at position 173(H)(right), (C)wild type amino acid at position 176 (G)(left)mutant type amino acid at position 176(W)(right).

We used DDMut to predict potential alterations in hydrogen bonds as consequences of the deleterious missense variants in the *MBP* gene. Our results indicated that the substitutions rs1971676214 (D173E) and rs772570115 (G176W) are likely to disrupt hydrogen bond formation, which could potentially affect the local conformation and stability of the MBP protein. In contrast, rs1242552448 (D173H) was not predicted to interfere with hydrogen bonding (S11 Table). The structural impact of these variants is displayed in Fig 9.

### 3.14. MBP—protein interaction and disease association

**Fig 10:**
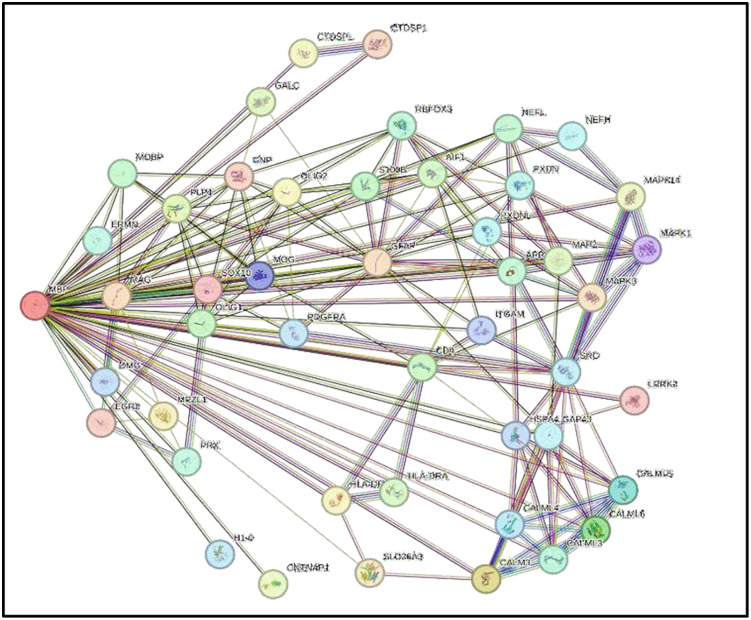
STRING protein–protein interaction network on MBP.

**Fig 11:**
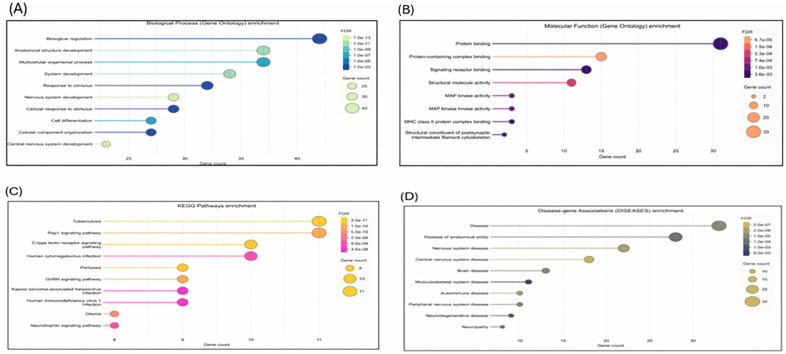
Enrichment analyses of MBP-interacting proteins identified through the STRING database. Gene Ontology enrichment for Biological Processes (A), Gene Ontology enrichment for Molecular Functions (B), KEGG pathway enrichment analysis (C), and Enrichment of Disease-Gene Associations (D).

STRING was used to analyze the wild-type MBP protein–protein interaction network and associated pathways. The network comprised 47 nodes and 178 observed edges, which is substantially higher than the 66 edges expected, with a PPI enrichment p-value < 1.0 × 10 ⁻ ¹⁶, indicating significant functional enrichment. MBP exhibited interactions with key myelin- associated proteins, including PLP1, MAG, MOG, MOBP, and CNP. MBP also interacted with transcription factors (SOX10, OLIG1, and OLIG2) and immune-related proteins, including CD4, ITGAM, and HLA class II molecules (HLA-DRA, HLA-DPB1). Additional connections were observed with cytoskeletal proteins (NEFL, GFAP), signaling molecules (MAPK1, MAPK3, SRC), and calcium-binding proteins (CALM1–CALM6) (Fig 10). We further studied the GO for MBP and its related proteins. We found that 45 interacting proteins were enriched in the Biological Process of biological regulation, which emerged as the most significantly enriched term (p-value < 0.05), encompassing the highest number of MBP-interacting proteins (Fig 11A). Within the GO Molecular Function category, protein binding was identified as the most significantly enriched function (p-value < 0.05) (Fig 11B). KEGG pathway enrichment analysis highlighted the RIG-I-like receptor signaling pathway and cytokine-cytokine receptor interaction among the most significantly enriched pathways (p-value < 0.05) (Fig 11C). Moreover, disease-gene association analysis revealed that nervous system diseases and central nervous system disorders were among the top enriched disease categories (Fig 11D).

In the present study, a comprehensive computational approach was employed to assess the potential pathogenicity of both noncoding and coding SNPs in the *MBP* gene. Expression analysis using GTEx data indicated that *MBP* is predominantly expressed in brain tissues (Fig 1), consistent with its established role as a structural component of the myelin sheath in the CNS. This tissue-specific enrichment underscores the functional specialization of *MBP* in neuronal myelination and the maintenance of axonal integrity. In this study we analyzed 2,709 SNPs within the 3′UTR (S1 Table) and 614 were nsSNPs (S2 Table). We identified 194 SNPs in the 3′UTR with RegulomeDB scores ≤ 2b (Fig 3). Functional evaluation using RegulomeDB enabled a systematic assessment of these noncoding variants, highlighting their likely impact on gene regulation. SNPs with lower scores (closer to 1) are more likely to affect the binding of regulatory proteins and impact gene expression. These findings contribute to our understanding of the complex interplay between genetic variations and gene function, potentially influencing disease susceptibility or other phenotypic traits. Consistent with the GTEx analysis, two 3′UTR variants (rs9199 and rs1048947) showed association with MBP expression in skeletal muscle, with small but detectable changes in normalized expression (Fig 4). These eQTLs were identified using nominal p-value thresholds without multiple-testing correction (Table 2) and should therefore be interpreted as exploratory, hypothesis-generating signals that require replication in independent datasets and functional validation Although *MBP* is classically regarded as a central nervous system–restricted gene with a primary role in myelin formation, previous studies have reported MBP immunoreactivity and the presence of MBP epitopes in non-neural tissues and cell types [42, 43]. The detection of an MBP eQTL in a non-CNS tissue is notable for the complexity of tissue- specific gene regulation. Potential explanation is that neural and muscle tissues possess a shared regulatory mechanism, wherein the expression of MBP is controlled by common transcriptional mechanisms or chromatin configurations. Additionally, the muscle tissue eQTLs for MBP could represent low level expression or regulatory activity of the gene in a specific context that is not fully captured in the GTEx data. These observations support the biological credibility of low-level or context-dependent MBP expression outside the nervous system. Nevertheless, given the comparatively limited expression of MBP in non-neural tissues, this finding should be interpreted with caution. Consistent with a potential regulatory role, both rs9199 and rs1048947 had RegulomeDB score of 1f, indicating a significant effect on regulatory annotation, however, experimental validation will be necessary to establish its functional impact and biological significance. Analysis of eight functionally significant and 3′UTR SNPs from RegulomeDB (rank ≤ 2b) provided insights into their potential regulatory effects on miRNA binding sites and mRNA structure (Table 3). These SNPs were specifically evaluated using RNAfold to compare MFE and secondary structure between wild-type and mutant sequences. Six out of eight functionally significant 3′ UTR variants had a change in MFE (Table 4) and altered mRNA structure (Fig 5), with three destabilizing and three stabilizing the transcript, while the remaining two showed minimal effect. These results underscore the value of combining regulatory scoring and structural predictions to prioritize variants with potential functional significance, as alterations in mRNA conformation or miRNA binding may influence gene expression through transcriptional or post-transcriptional mechanisms. Moreover, we predicted only 3 variants, rs1971676214 (D173E), rs1242552448 (D173H), and rs772570115 (G176W), as potential deleterious nsSNPs and destabilizing the protein structure using various bioinformatics tools (Table 7). Upon studying the population allele frequency of the three predicted disease-causing nsSNPs using the gnomAD database, we found only one nsSNP, rs1971676214 (D173E), was extremely rare in the global population, consistent with very rare variation. While rs1242552448 (D173H) and rs772570115 (G176W) were not reported in the gnomAD database. The rarity of these predicted deleterious nsSNPs in gnomAD emphasizes their uncharacterized but potentially disease-causing nature. Conservation and solvent accessibility analysis of MBP identified three nsSNPs, rs1971676214 (D173E), rs1242552448 (D173H), and rs772570115 (G176W), located in highly conserved, buried structural residues (Table 8), highlighting their potential functional and structural significance. These positions are essential for maintaining the tertiary structure, and mutations may destabilize MBP folding, disrupt its interaction network and impair biological function. These findings highlight the importance of integrating evolutionary conservation and structural context when evaluating the impact of nsSNPs on *MBP* and associated neurological disorders. AlphaFold model suggests that residues D173 and G176 lie in regions with relatively high local confidence (pLDDT ≈ 76–79), providing a reasonably defined backbone and side-chain environment for these positions (Fig 7). ConSurf scores of 9 indicate that these buried residues are highly conserved, consistent with the idea that even conservative substitutions at these sites may perturb local packing and longer-range structural organization. However, MBP has an intrinsically disordered nature, the AlphaFold model should be viewed as a plausible conformational ensemble rather than a fixed native structure, and any inferred local effects of the D173E, D173H, and G176W substitutions remain hypothesis-generating rather than definitive The result of DDMut analysis provides mechanistic insight by demonstrating that D173E and G176W but not D173H are likely to alter local hydrogen bonds and the ionic contacts (Fig 9). This pattern suggests that not all predicted destabilizing nsSNPs act through the same mechanisms and that some variants may affect packing or hydrophobic interactions rather than hydrogen bonding. STRING-based analysis revealed MBP as a central hub interacting with proteins involved in myelin formation, neural development, and immune regulation (Fig 10). Interactions with immune-related proteins, including MHC class II and CD4, underscore MBP’s immunogenic potential in autoimmune conditions, including multiple sclerosis.

Enrichment analysis indicated that biological regulation (Fig 11A) and protein binding (Fig 11B) are the most prominent functional categories, while KEGG pathways highlighted immune-related signaling, including RIG-I-like receptor and cytokine–cytokine receptor interactions (Fig 11C), relevant to CNS immune responses. Disease-gene analysis linked MBP-associated proteins to the nervous system and autoimmune disorders (Fig 11D), supporting its established role as a major autoantigen in MS and its involvement in neurodegenerative pathways. These findings emphasize MBP’s dual role in structural maintenance and immune-mediated neural processes. Importantly, none of the eight UTR SNPs or the three nsSNPs are documented in the ClinVar database, nor have they been reported in peer-reviewed literature to date, indicating that they may represent novel variants. All predictive algorithms consistently classify these substitutions as potentially deleterious, identifying them as strong candidates for future functional studies and experimental validation. Conclusion Our study provides insight into the effect of both noncoding and coding nsSNPs on the *MBP* gene, its protein 3D structure, and function. This study might be helpful in future studies of *MBP* to better understand its role in immunity, especially in MS. This study was carried out using computational tools and web-based servers. Given that all predictive algorithms consistently classify these variants as potentially deleterious, they constitute strong candidates for future functional characterization and epidemiological and experimental validation and do not provide evidence of clinical causality. Biophysical stability studies and T-cell epitope mapping studies with D173E, D173H, and G176W- MBP will play a crucial role in validating the computational results.

## Supporting information

S1 Table List of *MBP* 3′ UTR SNPs retrieved from NCBI. This table presents the 3′ UTR SNPs evaluated in this study.
(XLSX)

S2 Table List of nsSNPs identified in the *MBP* gene. This table presents nsSNPs subjected to functional analysis.
(XLSX)

S3.1 Table RegulomeDB scoring of *MBP* 3′ UTR SNPs. Regulatory scores for *MBP* 3′ UTR SNPs (SNP 1–135).
(XLSX)

S3.2 Table RegulomeDB scoring of *MBP* 3′ UTR SNPs. Regulatory scores for *MBP* 3′ UTR SNPs (SNP 136–247).
(XLSX)

S3.3 Table RegulomeDB scoring of *MBP* 3′ UTR SNPs. Regulatory scores for *MBP* 3′ UTR SNPs (SNP 248–356).
(XLSX)

S3.4 Table RegulomeDB scoring of *MBP* 3′ UTR SNPs. Regulatory scores for *MBP* 3′ UTR SNPs (SNP 357–446).
(XLSX)

S3.5 Table RegulomeDB scoring of *MBP* 3′ UTR SNPs. Regulatory scores for *MBP* 3′ UTR SNPs (SNP 447–517).
(XLSX)

S3.6 Table RegulomeDB scoring of *MBP* 3′ UTR SNPs. Regulatory scores for *MBP* 3′ UTR SNPs (SNP 518–600).
(XLSX)

S3.7 Table RegulomeDB scoring of *MBP* 3′ UTR SNPs. Regulatory scores for *MBP* 3′ UTR SNPs (SNP 601–692).
(XLSX)

S3.8 Table RegulomeDB scoring of *MBP* 3′ UTR SNPs. Regulatory scores for *MBP* 3′ UTR SNPs (SNP 693–788).
(XLSX)

S3.9 Table RegulomeDB scoring of *MBP* 3′ UTR SNPs. Regulatory scores for *MBP* 3′ UTR SNPs (SNP 789–884).
(XLSX)

S3.10 Table RegulomeDB scoring of *MBP* 3′ UTR SNPs. Regulatory scores for *MBP* 3′ UTR SNPs (SNP 885–971).
(XLSX)

S3.11 Table RegulomeDB scoring of *MBP* 3′ UTR SNPs. Regulatory scores for *MBP* 3′ UTR SNPs (SNP 972–1065).
(XLSX)

S3.12 Table RegulomeDB scoring of *MBP* 3′ UTR SNPs. Regulatory scores for *MBP* 3′ UTR SNPs (SNP 1066–1155).
(XLSX)

S3.13 Table RegulomeDB scoring of *MBP* 3′ UTR SNPs. Regulatory scores for *MBP* 3′ UTR SNPs (SNP 1156–1236).
(XLSX)

S3.14 Table RegulomeDB scoring of *MBP* 3′ UTR SNPs. Regulatory scores for *MBP* 3′UTR SNPs (SNP 1237–1286).
(XLSX)

S4 Table PolymiRTS analysis of *MBP* 3′ UTR SNPs. Predicted effects of SNPs on miRNA binding sites.
(XLSX)

S5 Table SIFT prediction results for *MBP* nsSNPs. This table reports the functional impact predictions generated by SIFT, identifying nsSNPs classified as deleterious or tolerated based on the SIFT Tolerance Index.
(XLSX)

S6 Table PolyPhen-2 prediction results for *MBP* nsSNPs. This table presents PolyPhen-2 predictions for *MBP* nsSNPs, classifying variants as benign, possibly damaging, or probably damaging based on the PolyPhen-2 score.
(XLSX)

S7 Table nsSNPs predicted as deleterious by multiple tools. This table includes nsSNPs that are consistently classified as damaging by SIFT, PolyPhen-2, and PROVEAN, independent functional prediction tools.
(XLSX)

S8 Table META-SNP predictions for *MBP* nsSNPs. This table presents the integrated pathogenicity assessment of *MBP* nsSNPs.
(XLSX)

S9 Table ESNPs&GO prediction results for *MBP* nsSNPs. This table summarizes disease associations and their reliability using ESNP&GO, with the reliability index.
(XLSX)

S10 Table Population allele frequency and clinical annotation of *MBP* nsSNPs. This table shows global allele frequencies and functional annotations for prioritized *MBP* nsSNPs.
(XLSX)

S11 Table Predicted effects of *MBP* nsSNPs on hydrogen bonding using DDMut. This table shows predicted changes in hydrogen bond interactions.(PDB).
(XLSX)

S1 File Structure file for the wild type MBP. This is a PDB structure file of the MBP using AlphaFold.
(PDB)

S2 File Structure file for D173E variant. This file shows the PDB structure of the D173E mutant protein.
(PDB)

S3 File Structure file for D173H variant. This file shows the PDB structure of the D173H mutant protein.
(PDB)

S4 File Structure file for G176Wvariant. This file shows the PDB structure of the G176W mutant protein.
(PDB)
